# Ability to cause erythema migrans differs between *Borrelia burgdorferi* sensu lato isolates

**DOI:** 10.1186/1756-3305-6-23

**Published:** 2013-01-22

**Authors:** Ellen Tijsse-Klasen, Nenad Pandak, Paul Hengeveld, Katsuhisa Takumi, Marion PG Koopmans, Hein Sprong

**Affiliations:** 1 Centre for Infectious Disease Control, National Institute for Public Health and the Environment (RIVM), P.O. Box 1, 3720BA, Bilthoven, Netherlands; 2 Department of Infectious Diseases, General Hospital “Dr. Josip Bencevic”, Slavonski Brod, Croatia; 3 Department of Virology, Erasmus Medical Centre, Rotterdam, Netherlands

**Keywords:** Lyme borreliosis, Erythema migrans, Molecular epidemiology, Virulence marker

## Abstract

**Background:**

Lyme borreliosis is a tick-borne disease caused by *Borrelia burgdorferi* sensu lato. The variety of characteristic and non-specific clinical manifestations is partially explained by its genetic diversity. We investigated the ability of *B*. *burgdorferi* sl isolates to cause erythema migrans.

**Methods:**

The genetic constellation of isolates from ticks was compared to isolates found in erythema migrans. PCR and sequence analysis was performed on the plasmid-encoded *ospC* and the chromosomal 5S-23S rDNA spacer region (IGS).

**Results:**

Seven different *B*. *burgdorferi* sl genospecies were identified in 152 borrelia isolates from ticks and erythema migrans biopsies. *B afzelii* (51%) and *B*. *garinii* (27%) were the most common in ticks. From the 44 sequences obtained from erythema migrans samples 42 were *B*. *afzelii*, one *B*. *garinii* and one *B*. *bavariensis*. Significant associations with erythema migrans formation were found for four IGS and two *ospC* types. Five from 45 *ospC* types were associated with more than one genospecies.

**Conclusions:**

*B*. *burgdorferi* sl isolates differ in their propensity to cause erythema migrans. These differences were also found within genospecies. In other words, although *B*. *afzelii* was mostly associated with erythema migrans, some *B*. *afzelii* isolates had a low ability to cause erythema migrans. Our data further support the occurrence of plasmid exchange between borrelia genospecies under natural conditions.

## Background

Lyme borreliosis is the most common tick-borne infectious disease in North America and in countries with moderate climates in Eurasia. Lyme borreliosis is caused by several members of the *Borrelia burgdorferi* sensu lato (sl) complex. Based on the genetic diversity, this large family of spirochetes has been subdivided into 19 different taxonomic entities or genospecies, of which several have been shown to cause disease in humans including *B*. *burgdorferi* sensu stricto (ss), *B*. *afzelli* and *B*. *garinii*[1]. Lyme borreliosis can present with a variety of typical clinical manifestations. An early sign of Lyme borreliosis is erythema migrans (EM), which presents as a circular expanding skin rash. In a later stage, infections can present with arthritis, carditis or acrodermatitis chronica atrophicans (ACA), as well as neurological symptoms, non-specific symptoms such as fatigue, generalized pain and cognitive complaints [2]. All late stages of Lyme borreliosis can occur without preceding EM.

It has been proposed that the variability in the clinical manifestations of Lyme borreliosis can be partly explained by the involvement of different *B*. *burgdorferi* sl genospecies [1,3,4]. Lyme arthritis and carditis have mainly been associated with *B*. *burgdorferi* ss, whereas *B*. *garinii* has been associated preferentially with neuroborreliosis [5]. Erythema migrans can be caused by several *B*. *burgdorferi* sl genospecies but *B*. *afzelii* seems to be overrepresented in this group [6]. *B*. *afzelii* is also the genospecies most commonly associated with ACA, although *B*. *garinii* and *B*. *burgdorferi* ss have also been isolated from ACA cases [6,7]. Even within genospecies, differences in pathogenicity between *B*. *burgdorferi* ss strains have been shown [8-10]. However, despite numerous attempts, the various manifestations of Lyme borreliosis in humans cannot conclusively be attributed to infections with one specific genospecies of *B*. *burgdorferi* sl [11-14]. Understanding the possible differential pathogenicity between and within different borrelia genospecies is important because it will improve understanding of disease etiology and facilitate laboratory diagnostics.

Here, we investigated whether different genospecies and different members within a genospecies differ in their propensity to cause a typical manifestation of Lyme borreliosis, namely EM. The relative abundance of different *B*. *burgdorferi* sl genotypes in EM skin samples does not only depend on their ability to cause EM, but also on their relative abundance in the tick population. Therefore, the genetic divergence of genospecies found in EM skin samples was compared to the genetic divergence of *Borrelia* in local tick populations. We investigated which typing methods could predict EM formation best: genospecies determination, IGS- or *ospC* haplotyping.

The rrfA-rrlB (5S-23S) rDNA intergenic spacer (IGS) has been used extensively to identify *B*. *burgdorferi* sl genospecies around the world and provides a good resolution within genospecies [15-20]. While IGS is a suitable marker to differentiate genospecies, unlike other, mainly surface exposed genes, it is not a virulence gene [21]. Outer surface protein C (*ospC*), which is encoded on the cp26 plasmid, is the best studied of these [22,23]. Although its exact function is still unknown, it has been shown to play an important role in early infection [22,24]. Several studies from the United States found that only a selection of *ospC* types found in ticks are also detected in patient material, especially in disseminated infections [8-10]. More recent studies, however, have extended the number of pathogenic *ospC* genotypes substantially [25,26]. The involvement of *ospC* in early Lyme borreliosis makes it an interesting marker for studying the *Borrelia* genotypes involved in EM and *ospC* types might be more strongly correlated with EM than IGS types.

## Methods

### Collection of skin biopsies and serum samples

During the period 2008-2011, patients presenting in one of the three participating hospitals in Slavonski Brod, Rijeka or Čakovec with erythema migrans (EM) were enrolled in the study. EM was diagnosed by experienced physicians and each patient underwent skin biopsy of the EM active edge. The area of the biopsy was infiltrated with 1.5 ml of 1% lidocain hydrochloride solution after which the incisional skin biopsy was performed. Skin samples were stored in 70% ethanol at –20°C until further analysis. The study was approved by the General Hospital Slavonski Brod Ethic Committee and all patients signed an informed consent form.

### Collection of ticks

**Figure 1:**
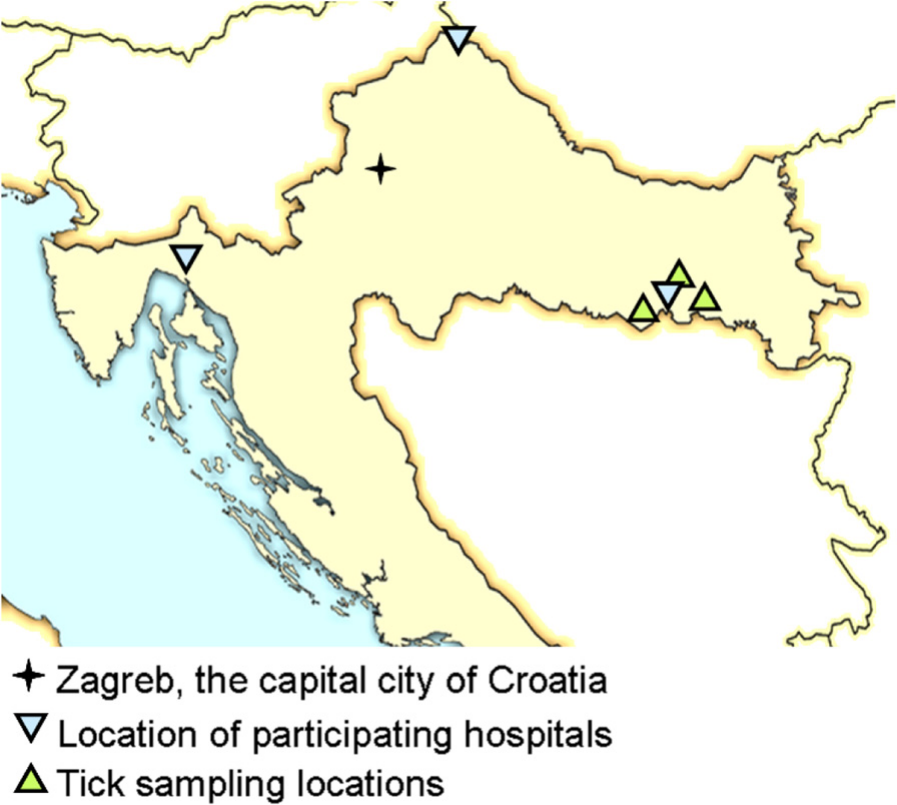
Map of Croatia with sampling sites indicated.

During the period March-June 2011, ticks were collected at three different sites located 15 to 20 km east, north and west of Slavonski Brod city limits (Figure 1). Ticks were collected by blanket dragging, placed in individual tubes and stored at –20°C until analysis.

### DNA extraction

DNA of skin biopsies was extracted using the DNeasy Blood and Tissue Kit (Qiagen, Hilden, Germany) protocol for animal tissue according to the manufacturer’s instructions.

DNA from vegetation ticks was extracted by alkaline lysis as described earlier [27]. If PCR inhibition was observed in conventional PCR, samples were further purified. Supernatant or the original tick was used to extract DNA using a DNeasy Blood and Tissue kit (Qiagen, Hilden, Germany) according to the manufacturer’s protocols for body fluids or engorged ticks, respectively.

### Detection and identification of *B*. *burgdorferi* sl

*B*. *burgdorferi* sl was detected using a duplex qPCR based on the OspA and flagellin genes and carried out on a LightCycler 480 instrument (Roche Diagnostics Nederland B.V, Almere, the Netherlands) [28]. Reactions were done in a final volume of 20 μl with iQ multiplex Powermix, 3 μl of template DNA and 0.2 μM for all primers and probes (Table 1) except for OspA_probe, which was used at a final concentration of 0.1 μM. After iTaq DNA polymerase activation at 95°C for 5 minutes, 60 cycles of 95°C for 5 seconds, 60°C for 35 seconds (ramp rate 2,2°C/s and a single point measurement at 60°C) 37°C for 20 seconds were run. Quantification cycle (Cq) values were calculated with the LightCycler 480 software (release 1.5.0. SP3). Positive controls (*B*. *burgdorferi* ss B31) and negative water controls were used on every plate tested. Samples positive for one or both targets were scored positive for *B*. *burgdorferi* sl.

**Table 1:** DNA sequences of primer and probes

**Oligo name**	**Sequence (5’-3’)**
Duplex qPCR	
OspA_F	AATATTTATTGGGAATAGGTCTAA
OspA_R	CTTTGTCTTTTTCTTTRCTTACAAG
FlaB-F	CAGAIAGAGGTTCTATACAIATTGAIATAGA
FlaB-Rc/t	GTGCATTTGGTTAIATTGYGC
OspA_probe	FAM-AAGCAAAATGTTAGCAGCCTTGA-BHQ-1™
FlaB-probe	JOE-CAACTIACAGAIGAAAXTAAIAGAATTGCTGCTGAICA
IGS PCR	
5S	GAGTTCGCGGGAGAGTAGGTTATTGCC
23S	TCAGGGTACTTAGATGGTTCACTTCC
OspC PCR	
OC6	AAAGAATACATTAAGTGCGATATT
OC602	GGGCTTGTAAGCTCTTTAACTG

FAM: 6-carboxyfluorescein; JOE: -carboxy-4',5'-dichloro-2',7'-dimethoxyfluorescein.

qPCR positive samples were subjected to conventional PCR on the 5S-23S rRNA (rrfA-rrlB) intergenic spacer region (IGS) and *ospC* gene. IGS PCR was done using primers 5S and 23S (Table 1) at final concentrations of 0.5 μM. The PCR was performed in a total reaction volume of 25 μl using HotStarTaq master mix (Qiagen, Hilden, Germany) under following conditions: 15 min 94°C, then cycles of 20 s 94°C, 30 s 70°C, 30 s 72°C lowering the annealing temperature 1°C each cycle until reaching 60°C, then 40 cycles at this annealing temperature and a final elongation step for 7 min at 72°C.

Conventional PCR on the *ospC* gene was performed using primers OC6 and OC602 at final concentrations of 0.4 μM [29]. The PCR was performed using HotStarTaq master mix under following conditions: 15 min 94°C, then cycles of 30 s 94°C, 30 s 60°C, 50 s 72°C lowering the annealing temperature 1°C each cycle until reaching 50°C, then 35 cycles at an annealing temperature of 53°C and a final elongation step for 7 min at 72°C.

Amplicons from conventional PCRs were sequenced on both strands on a 3730 DNA Analyzer using ABI PRISM Big-Dye Terminator Cycle sequencing Ready Reaction kit (Applied Biosystems, Carlsbad, CA, USA).

*B*. *burgdorferi* sl genospecies were inferred from the IGS sequence by comparing the sequences with a large local database (± 3000 entries) including, amongst others, all IGS entries from GenBank and 170 IGS sequences from different samples confirmed by multi-locus sequence typing.

In order to minimize contamination, PCR proceedings were performed in three separate rooms, of which the DNA extraction room was kept at negative pressure and the reagent setup and sample addition rooms were kept at positive pressure. All rooms had airlocks.

### Genetic analysis

Sequence quality was visually checked and sequences of both strands were assembled using Bionumerics software. IGS sequences were aligned together with a *B*. *afzelii* reference sequence from Genbank (strain PKo, Genbank entry CP002933). An IGS or *ospC* type was defined as a sequence diverging at least one nucleotide or gap from any other sequence in the dataset. The number of IGS types was determined based on the sequence fragment between position 438843 and 439146 of whole genome sequence of this reference strain. Likewise, *ospC* sequences were aligned with a *B*. *afzelii* PKo reference strain (Genbank entry CP002934) between position 17065 and 17463 and *ospC* types were identified. Short or poor quality *ospC* sequences, of which some might have been caused by double infections or multiple plasmids, were rejected from further analysis. IGS and *ospC* types were numbered independent from their phylogenetic relationships.

### Statistical analysis

The diversity of IGS and *ospC* types found in ticks was compared with that in EM skin samples using a binomial test of statistical significance. The hypothesis that certain IGS or *ospC* types are found equally often in EM skin samples and in tick samples was compared with the alternative that a certain IGS or *ospC* type is predominantly associated with one of the sample types. A likelihood ratio test with a degree of freedom of one was performed on this model. A certain IGS or *ospC* type is associated with ticks or EM skin samples if the *p*-value of the likelihood ratio test was less than 0.05.

## Results

### Collected samples

During a four-year period, 67 patients presenting in one of the three participating hospitals were enrolled in the study. Forty-one DNA isolates from EM biopsies were taken at a hospital in Slavonski Brod, 16 in Čakovec and 10 in Rijeka. In the same period, 1573 ticks were collected for molecular analysis from three locations near Slavonski Brod (Figure 1). Of these, 1433 (91%) were identified as adult *Ixodes ricinus*, 117 (7.4%) as *Dermacentor marginatus* and 24 (1.5%) as *Haemaphysalis concinna*.

### Detection and identification of *B*. *burgdorferi* sl

Using the duplex *B*. *burgdorferi* sl qPCR, 254 of 1432 *I*. *ricinus* ticks (18%) were found positive for *B*. *burgdorferi* sl. All *D*. *marginatus* (n = 117) and *H*. *concinna* ticks (n = 24) were negative. *B*. *burgdorferi* sl positive *I*. *ricinus* were subjected to conventional PCR on both IGS and *ospC* followed by sequencing. Due to the lower sensitivity of conventional PCR compared to qPCR, only 108 IGS and 83 *ospC* sequences were obtained from qPCR positive ticks. From 67 skin samples 47 (70%) were found positive in duplex qPCR. Conventional PCR and sequencing on these samples yielded 44 IGS and 37 *ospC* sequences, respectively.

*Borrelia* genospecies were identified based on IGS sequences. Fifty-five of 108 sequenced isolates from ticks and 42 of 47 isolates from EM samples were identified as *B*. *afzelii*. Likewise, *B*. *garinii* was detected in 29 *I*. *ricinus* and one EM skin sample. *B*. *bavariensis* was only found in a single EM skin sample from Rijeka while isolates belonging to *B*. *burgdorferi* ss, *B*. *valaisiana*, *B*. *lusitianae* and *B*. *spielmanii* were only found in ticks (n = 5, 8, 10 and 1, respectively) (Table 2).

**Table 2:** **Number of isolates from tick and EM samples and IGS and *OspC *types distribution across genospecies**

**Borrelia species**	**Isolates from**		**Haplotypes of**	
	**Ticks (n)**	**EM (n)**	**IGS (#)**	**OspC (#)**
*B. afzelii*	55	42	16	23
*B. garinii*	29	1	7	11
*B. burgdorferi ss*	5	0	3	2
*B. valaisiana*	8	0	2	5
*B. bavariensis*	0	1	1	**
*B. lusitaniae*	10	0	1	5
*B. spielmanii*	1	0	1	1
Total	108	44	31	47 (45*)

n: number of isolates; #: number of haplotypes. * Some OspC haplotypes were found in several genospecies, therefore entries in this column do not add up to 45. ** OspC could not be determined.

In the following, the term isolate is used to describe a DNA sample isolated from a human or tick. This implies that an isolate may contain more than one *B*. *burgdorferi* sl genospecies.

**Figure 2:**
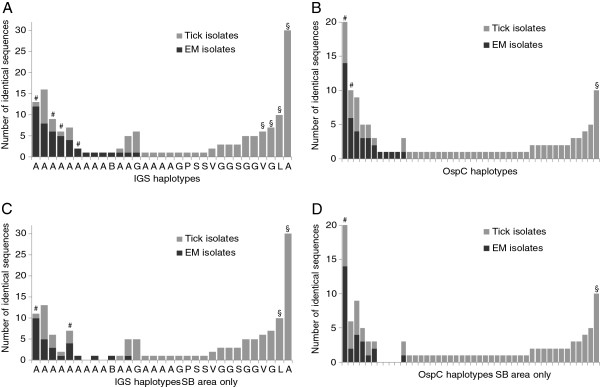
**Distribution of IGS and OspC types across EM skin samples and ticks.** Figure **A** shows distributions of IGS types, and figure **B** that of OspC types in EM samples and ticks. Figure **C** and **D** show the same analyses as **A** and **B** with exclusion of EM samples from locations Rijeka and Čakovec. Letters under x-axis of **A** and **C** indicate genospecies (V: *B*. *valaisiana*, L. *B*. *lusitianiae*, A: *B*. *afzelii*, G: *B*. *garinii*, B: *B*. *bavariensis*, P: *B*. *spielmanii*, S: *B*. *burgdorfe*ri s.s.). Signs above bars indicate significant differences between tick and EM samples: §: more often in ticks, #: more often in EM.

In total, IGS alignment revealed 31 different IGS types, with each type diverging at least one nucleotide or gap from other sequences in the sample (Additional file 1: Figure S1). *B*. *afzelii* isolates in this study could be subdivided in 16 IGS types. Seven, three and two different IGS types were found for *B*. *garinii*, *B*. *burgdorferi* ss and *B*. *valaisiana*, respectively, while the remaining genospecies (*B*. *bavariensis*, *B*. *lusitaniae*, *B*. *spielmanii*) comprised a single IGS type. Four IGS types were exclusively found in EM samples, 18 only in tick samples and 9 in both sample types (Figure 2).

*OspC* alignment revealed 45 different *ospC* types (Additional file 2: Figure S2) of which four were found exclusively in EM skin samples, 34 only in tick samples and 7 in both sample types.

**Figure 3:**
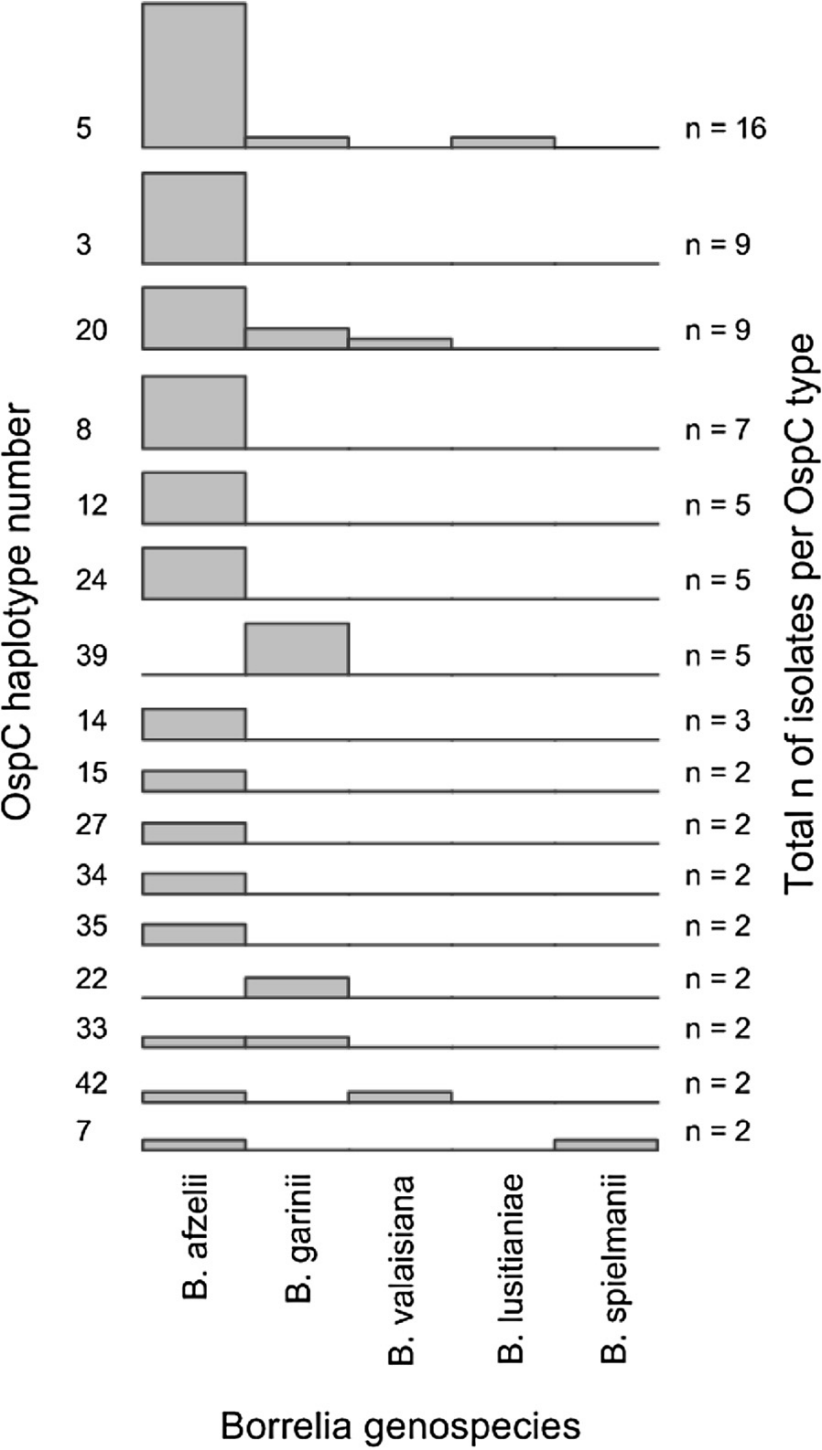
**Some OspC types are associated with more than one *B. burgdorferi *genospecies, indicating horizontal gene transfer.** Each graph represents one OspC type. Genospecies were inferred from IGS sequences. Only OspC types that were found more than once are shown.

In 66 samples from ticks and 33 samples from EM both IGS and *ospC* were sequenced. Analysis of these samples showed that five *ospC* types (numbers 5, 20, 33, 42 and 7; Figure 3) were found in two or more genospecies.

### Correlation of *B*. *burgdorferi* sl IGS and *ospC* types with EM

Only 13 of 31 IGS types that were identified in this study were detected in skin biopsies from erythema migrans patients. The skin samples in this study were collected in three different locations throughout Croatia (Slavonski Brod, Rijeka and Čakovec) but ticks were only collected in proximity to Slavonski Brod. Considering all collected skin samples, IGS types 21, 18, 6 and 7 (order as in Figure 2A) were found significantly more often in patient samples than would have been expected on basis of the *Borrelia* types found in ticks. In contrary, IGS types 2, 14, 3 and 25 were significantly less often found in EM skin samples. When only samples from Slavonski Brod were used, two IGS types remained significantly associated with EM (Figure 2B) and two were found significantly less often in EM than expected.

The second genetic marker used in this study, *ospC*, is encoded on a plasmid of *B*. *burgdorferi*. Of the 44 different *ospC* types that were detected, 24 were found in *B*. *afzelii* but not necessarily restricted to this genospecies. There was a strong correlation of *ospC* type with EM. This correlation was significant for *ospC* types 5 and 3 (order as in Figure 2B) and types 39 and 20 were found significantly less often than expected. These associations stayed significant for *ospC* types 5 and 20 when only skin samples from Slavonski Brod were analysed (Figure 2D).

## Discussion

Three tick species were collected during this study, all of which had been reported from the northern Balkan region before [30]. The majority of the ticks collected were *I*. *ricinus* which is the main vector for Lyme borreliosis in Europe. The infection rates of *I*. *ricinus* were similar to those reported in earlier studies in Croatia and other European countries [31-33] but much higher and lower infection rates were also reported from a neighbouring country [34,35]. Seven out of nine *B*. *burgdorferi* sl genospecies known to occur in Europe were identified and only *B*. *bissettii* and *B*. *kurtenbachii* were not found [1]. As in most European countries *B*. *afzelii* was the most common genospecies identified.

In EM skin biopsies three different *B*. *burgdorferi* sl genospecies were detected and the previously recognized overrepresentation of *B*. *afzelii* in the skin condition erythema migrans was confirmed [4,6]. More importantly, a strong correlation between certain IGS types within the *B*. *afzelii* cluster and EM were found.

In earlier studies, certain *ospC* types of *B*. *burgdorferi* ss have been shown to be associated with higher probabilities of invasiveness and human disease [8-10,36]. Lagal and co-workers found similar associations for *B*. *afzelii* and *B*. *garinii* strains in Europe but did not compare the frequency of the different *ospC* types in clinical samples to those in the tick population [37]. We expanded these observations to EM in Croatia, where a broad range of *B*. *burgdorferi* sl is present and supported our findings with substantial tick data. Correlations of EM with certain types of the plasmid-encoded marker *ospC* were evident from our datasets. The expectation that this gene, due to its role in early infection, might show a stronger correlation to EM than IGS, could not be confirmed [24].

IGS type determination predicted the isolates propensity to cause EM better than genospecies determination alone. This is illustrated by the example that both the isolates with the highest and lowest propensity to cause EM belonged to *B*. *afzelii* genospecies. Significant positive and negative association with EM were more often found for IGS- than for *ospC*-typing (Figure 2) suggesting that IGS predicts EM formation better than *ospC*. This would imply that at least some of the genes responsible for EM formation are located on the chromosome or other non-exchangeable genomic elements.

As pointed out by Dykhuizen and co-workers it is important to use tick data alongside patient data to correctly estimate the propensity of strains to cause infection [36]. This was done in the present study by sampling ticks around the main study site, from which the majority of patient samples were obtained. As a geographic bias could not be excluded, analyses were done for the whole study population or the Slavonski Brod study population alone. When only Slavonski Brod samples were taken into account, fewer IGS and *ospC* types were significantly more or less often found in patients than expected than when the whole study population was used. However, even when only isolates from this site were analysed one *ospC* and two IGS types were significantly more often found in patients than expected. Four patients from Čakovec and Rijeka study areas had *B*. *burgdorferi* sl sequences which were not found in any of the ticks can be explained by the lack of tick data from those study areas. These data highlight the importance of tick sampling representative for a certain study population. As Lyme borreliosis can be acquired by bites from adult as well as nymphal *I*. *ricinus*, a further potential bias lies in the testing of adult ticks only. Adult ticks might have obtained *B*. *burgdorferi* sl during feeding as nymphs. Hosts that fed nymphs and larvae might slightly differ and thus adult ticks might be infested with a different subpopulation of *B*. *burgdorferi* sl than nymphs.

It is unlikely that the propensity to cause EM or the invasiveness of *Borrelia* strains is determined by a single virulence factor alone. We rather hypothesize that a combination of different virulence factors encoded on the chromosome as well as on several plasmids of *Borrelia* determine the pathogenicity and invasiveness of different strains. Recently, a study showed that expression of *ospC* is regulated by a gene on a different plasmid [38]. This emphasizes that the virulence of a certain strain, but also its fitness in a certain ecosystem, is a product of a complex system of genes interacting with each other and that it might be influenced by horizontal gene transfer. Even with the limited number of samples (n = 99) for which both genetic markers were available, five *ospC* types were found in more than one genospecies. This shows that substantial horizontal gene transfer takes place between different genospecies. Plasmid transfer has been reported before and shows that the apparent clonality seen in North American *B*. *burgdorferi* ss is lacking in European genospecies [10,39-41].

The current study shows that different *Borrelia* IGS and *ospC* types exhibit different propensities to cause EM. We cannot exclude, however, that the genotypes involved in EM differ by location and in time. Furthermore, *B*. *burgdorferi* sl with IGS and *ospC* types identified to have a low propensity to cause EM here, could omit this early manifestation of Lyme borreliosis and directly cause disseminated manifestations of the disease. Besides the genetic constitution of *B*. *burgdorferi* sl, other explanations for the variability in the clinical manifestations of Lyme borreliosis are the (genetic) constitution of the human host [42,43], co-infections with other pathogens [44-46] and the bacterial load of ticks. However, the current study only focuses on genetic aspects of *B*. *burgdorferi* sl.

In future studies other sample types, such as cerebrospinal fluid or arthritis isolates, could be analysed in a similar way as was done here. The two markers used here are not directly responsible for EM formation and efforts should be undertaken to identify virulence factors that determine clinical manifestations. Once virulence genes are identified, risk assessment for Lyme borreliosis could be improved by including only human pathogenic *B*. *burgdorferi* sl into the risk assessment. In future, this information might help in the design of ecological interventions to reduce Lyme borreliosis risk, the improvement of serology-based diagnosis by use of more specific antigens or for the development of specialised vaccines targeting virulent *B*. *burgdorferi* sl strains.

## Conclusion

We identified a strong correlation of certain IGS and *ospC* types with formation of erythema migrans. This indicates that only a small subset of *B*. *burgdorferi* sl carried by ticks in the wild actually cause EM. Other genotypes either may be apathogenic for humans or may cause Lyme borreliosis without the typical first sign of infection.

However, as we restricted our investigation to an early form of Lyme borreliosis, it cannot be excluded that other *B*. *burgdorferi* sl genospecies and genotypes, not associated with EM, might omit this early sign of Lyme borreliosis and still lead to disseminated infections. This should be investigated using a broader range of patient materials from a broader geographic area.

## Competing interests

The authors declare that they have no competing interests.

## Authors’ contributions

NP and HS designed the study and NP collected samples. ETK and PH carried out laboratory experiments. ETK, KT and HS conducted genetic and statistic data analyses. ETK, HS and MK wrote the final manuscript. All authors read and approved the final version of the manuscript.

## Supplementary Material

Additional file 1: Figure S1 Alignment of all identified IGS haplotypes with B. afzelii PKo (CP002933), REGION: 438843-439146. Click here for file

Additional file 2: Figure S2 Alignment of all identified OspC haplotypes with B. afzelii PKo (CP002934), REGION: 17065-17463. Click here for file
