# Supplementary material for: Ability to cause erythema migrans differs between Borrelia burgdorferi sensu lato isolates
Source: Parasit Vectors. 2013 Jan 22;6:23. doi: 10.1186/1756-3305-6-23 (PMC3599126; doi:10.1186/1756-3305-6-23)
Supplement: Additional file 1: Figure S1 — Alignment of all identified IGS haplotypes with B. afzelii PKo (CP002933), REGION: 438843-439146. [file 1756-3305-6-23-S1.pdf]

Supplementary figure 1: Alignment of all identified IGS haplotypes with B. afzelii PKo (CP002933), REGION: 438843-439146

```
          .30       .40       .50       .60       .70       .80       .90       .100      .110      .120      .130
PKo  TATCGCCTCTATTATTT--AGATAATAGATAGCTAGCATCTTGCTAGCTGGATTACTCCATTCGGTAATCTTGGGATCAATAAATGTTTGCTTAT-CCCCCAAGCTTTTC
0001 TATCGCCTCTATTATTT--AGATAATAGATAACTAGCATCTTACTAGTTGGATTACTCCATTCGGTAATCTTGGGATCAATAAATGTTTGCTTCT-CCCCCAAGCTTTTC
0002 TATCGCCTCTATTATTT--AGATAATAGATAACTAGCATCTTGCTAGTTGGATTACTCCATTCGGTAATCTTGGGATCAATAAATGTTTGCTTCT-CCCCCAAGCTTTTC
0003 TATCGCCTCTATTA-TT--AAATAATAGATAGCTAGCATCTTGCTAGCTGGATTACTCCATTCGGTAATCTTGGGATCAATAAATGTTTGCTTCT-CCCCCAAGCTTTTC
0004 TATCGCCTCTATTATTT--AGATAATAGATAGCTAGTATCTTGCTAGCTGGATTACTCCATTCGGTAATCTTGGGATCAATAAATGTTTGCTTCT-CCCCCAAGCTTTTC
0005 TATCGCCTCTATTATTT--AGATAATAGATAGCTAGCATCTTGCTAGCTGGATTACTCCATTCGGTAATCTTGGGATCAATAAATGTTTGCTTCT-CCCCCAAGCTTTTC
0006 TATCGCCTCTATTATTT--AGATAATAGATAGCTAGCATCTTGCTAGCTGGATTACTCCATTCGGTAATCTTGGGATCAATAAATGTTTGCTTTT-CCCCCAAGCTTTTC
0007 TATCGCCTCTATTATTT--AGATAATAGATAGCTAGCATCTTGCTAGCTGGATTACTCCATTCGGTAATCTTGGGATCAATAAATGTTTGCTTCTCCCCCCAAGCTTTTC
0008 TATCGCCTCTATTATTT--AGATAATAGATAGCTAGCATCTTGCTAGCTGGATTACTCCATTCGGTAATCTTGGGATCAATAAATGTTTGCTTCT-CCCCCAAGCTTTTC
0009 TATCGCCTCTATTATCT--AAATAATAGATAGCTAGCATCTTGCTAGCTGGATTACTCCATTCGGTAATCTTGGGATCAATAAATGTTTGCTTTT-CCCCCAAGCTTTTC
0010 TATCGCCTCTATTATTT--AAATAATAGATAGCTAGCATCTTGCTAGCTGGATTACTCCATTCGGTAATCTTGGGATCAATAAATGTTTGCTTCT-CCCCCAAGCTTTTC
0011 TATCGCCTCTATTATTT---AATAATAGATAGCTAGCATCTTGCTAGCTGGATTACTCCATTCGGTAATCTTGGGATCAATAAATGTTTGCTTCT-CCCCCAAGCTTTTC
0012 TATCGCCTCTATTATTT--AGATAATAGATAGCTAGCATCTTGCTAGCTGGATTACTCCATTCGGTAATCTTGGGATCAATAAATGTTTGCTTCT-CCCCCAAGCTTTTC
0013 TATCGCCTCTATTATTT--AAATAATAGATAGCTAGCATCTTGCTAGCTGGATTACTCCATTCGGTAATCTTGGGATCAATAAATGTTTGCTTCT-CCCCCAAGCTTTTC
0014 TATCGCCTCTATTATTT---AATAATAGATAGCTAGCATCTTGCTAGCTGGATTACTCCATTCGGTAATCTTGGGATCAATAAATGTTTGCTTCT-CCCCCAAGCTTTTC
0015 TATCGCCTCTATTATTT--AAATAATAGATAGCTAGCATCTTGCTAGCTGGATTACTCCATTCGGTAATCTTGGGATCAATAAATGTTTGCTTCT-CCCCCAAGCTTTTC
0016 TATCGCCTCTATTATTT--AAATAATAGATAGCTAGCATCTTGCTAGCTGGATTACTCCATTCGGTAATCTTGGGATCAATAAATGTTTGCTTCT-CCCCCAAGCTTTTC
0017 TATCGCCTCTATTATTT--AGATAATAGATAGCTAGCATCTTGCTAGCTGGATTACTCCATTCGGTAATCTTGGGATCAATAAATGTTTGCTTCT-CCCCCAAGCTTTTC
0018 TATCGCCTCTATTATTT--AGATAATAGATAGCTAGCATCTTGCTAGCTGGATTACTCCATTCGGTAATCTTGGGATCAATAAATGTTTGCTTCT-CCCCCAAGCTTTTC
0019 TATCGCCTCTATTATTT--AGATAATAGATAGCTAGCATCTTGCTAGCTGGATTACTCCATTCGGTAATCTTGGGATCAATAAATGTTTGCTTCTCCCCCCAAGCTTTTC
0020 TATCGCCTCTATTATTT--AGATAATAGATAGCTAGCATCTTGCTAGCTGGATTACTCCATTCGGTAATCTTGGGATCAATAAATGTTTGCTTAT-CCCCCAAGCTTTTC
0021 TATCGCCTCTATTATTT--AGATAATAGATAGCTAGCATCTTGCTAGCTGGATTACTCCATTCGGTAATCTTGGGATCAATAAATGTTTGCTTCTCCCCCCAAGCTTTTC
0022 TATCGCCTCTATTATTT--AGATAATAGATAGCTAGCATCTTGCTAGCTGGATTACTCCATTCGGTAATCTTGGGATCAATAAATGTTTGCTTCTCCCCCCAAGCTTTTC
0023 TATCGCCTCTATTATTT--AGATAATAGATAGCTAGCATCTTGCTAGCTGGATTACTCCATTCGGTAATCTTGGGATCAATAAATGTTTGCTTCT-CCCCCAAGCTTTTC
0024 TATCGCCTCTATTATTT--AGATAATAGATAGCTAGCATCTTGCTAGCTGGATTACTCCATTCGGTAATCTTGGGATCAATAAATGTTTGCTTCT-CCCCCAAGCTTTTC
0025 TATCGCCTCTATTATTT--AGATAATAGATAGCTAGCATCTTGCTAGCTGGATTACTCCATTCGGTAATCTTGGGATCAATAAATGTTTGCTTTT-CCCCCAAGCTTTTC
0026 TATCGCCTCTATTATTT--AGATAATAGATAGCTAGCATCTTGCTAGCTGGATTACTCCATTCGGTAATCTTGGGATCAATAAATGTTTGCTTTTCCCCCCAAGCTTTTC
0027 TATCGCCTCTATTATTTAGAGATAATAGATAGCTAGCATCTTGCTAGCTGGATTACTCCATTCGGTAATCTTGGGATCAATAAATGTTTGCTTTT-CCCCCAAGCTTTTC
0029 TATCGCCTCTATTATTT--AGATAATAGATAGCTAGCATCTTGCTAGCTGGATTACTCCATTCGGTAATCTTGGGATCAATAAATGTTTGCTTCT-CCCCCAAGCTTTTC
0030 TATCGCCTCTATTATTT--AAATAATAGATAGCTAGCATCTTGCTAGCTGGATTACTCCATTCGGTAATCTTGGGATCAATAAATGTTTGCTTCT-CCCCCAAGCTTTTC
0031 TATCGCCTCTATTATTT--AAATAATAGATAGCTAGCATCTTGCTAGCTGGATTACTCCATTCGGTAACCTTGGGATCAATAAATGTTTGCTTCT-CCCCCAAGCTTTTC
0032 ----------ATTATCT--AAATAATAGATAGCTAGCATCTTGCTAGCTGGATTACTCCATTCGGTAATCTTGGGATCAATAAATGTTTGCTTCT-CCCCCAAGCTTTTC
```

```
        .140      .150      .160      .170      .180      .190      .200      .210      .220      .230      .240
PKo  GCAGCTTACCACGACCTTCTTCGCCTTAAAGCTCCTAGGCATCCACCATAGACTCTTATTACTTTGACCATATTTTTATCTTCCATCTCTATTTTGCCAATTTGTTTATA
0001 GCAGCTTACCACGACCTTCTTCGCCTTAAAGCTCCTAGGCATTCACCATAGACTCTTATTACTTTGACCATATTTTTATCTTCCATCTCTATTTTGCCAATTTGTTTATG
0002 GCAGCTTACCACGACCTTCTTCGCCTTAAAGCTCCTAGGCATTCACCATAGACTCTTATTACTTTGACCATATTTTTATCTTCCATCTCTATTTTGCCAATTTGTTTATG
0003 GCAGCTTACCACGACCTTCTTCGCCTTAAAGCTCCTAGGCATTCACCATAGACTCTTATTACTTTGACCATATTTTTATCTTCCATCTCTACTTTGCCAATTTGTTTATG
0004 GCAGCTTACCACGACCTTCTTCGCCTTAAAGCTCCTAGGCATTCACCATAGACTCTTATTACTTTGACCATATTTTTATCTTCCATCTCTATTTTGCCAATTTGTTCATA
0005 GCAGCTTACCACGACCTTCTTCGCCTTAAAGCTCCTAGGCATTCACCATAGACTCTTATTACTTTGACCATATTTTTATCTTCCATCTCTATTTTGCCAATTTGTTCATA
0006 GCAGCTTACCACGACCTTCTTCGCCTTAAAGCTCCTAGGCATTCACCATAGACTCTTATTACTTTGACCATATTTTTATCTTCCATCTCTATTTTGCCAATTTGTTTATA
0007 GCAGCTTACCACGACCTTCTTCGCCTTAAAGCTCCTAGGCATCCACCATAGACTCTTATTACCTTGACCATATTTTTATCTTCCATCTCTATTTTGCCAATTTGTTTATA
0008 GCAGCTTACCACGACCTTCTTCGCCTTAAAGCTCCTAGGCATCCACCATAGACTCTTATTACTTTGACCATATTTTTATCTTCCATCTCTATTTTGCCAATTTGTTTATA
0009 GCAGCTTACCACGACCTTCTTCGCCTTAAAGCTCCTAGGCATCCACCATAGACTCTTATTACTTTGACCATATTTTTATCTTCCATCTCTATTTTGCCAATTTGTTTATA
0010 GCAGCTTACCACGACCTTCTTCGCCTTAAAGCTCCTAGGCATTCACCATAGACTCTTATTACTTTGACCATATTTTTATCTTCCATCTCTATTTTGCCAATTTGTTTATA
0011 GCAGCTTACCACGACCTTCTTCGCCTTAAAGCTCCTAGGCATTCACCATAGACTCTTATTACTTTGACCATATTTTTATCTTCCATCTCTATTTTGCCAATTTGTTTATA
0012 GCAGCTTACCACGACCTTCTTCGCCTTAAAGCTCCTAGGCATTCACCATAGACTCTTATTACTTTGACCATATTTTTATCTTCCATCTCTATTTTGCCAATTTGTTTATA
0013 GCAGCTTACCACGACCTTCTTCGCCTTAAAGCTCCTAGGCATTCACCATAGACTCTTATTACTTTGACCATATTTTTATCTTCCATCTCTATTTTGCCAATTTGTTTATA
0014 GCAGCTTACCACGACCTTCTTCGCCTTAAAGCTCCTAGGCATTCACCATAGACTCTTATTACTTTGACCATATTTTTATCTTCCATCTCTATTTTGCCAATTTGTTTATA
0015 GCAGCTTACCACGACCTTCTTCGCCTTAAAGCTCCTAGGCATTCACCATAGACTCTTATTACTTTGACCATATTTTTATCTTCCATCTCTATTTTGCCAATTTGTTTATA
0016 GCAGCTTACCACGACCTTCTTCGCCTTAAAGCTCCTAGGCATTCACCATAGACTCTTATTACTTTGACCATATTTTTATCTTCCATCTCTATTTTGCCAATTTGTTTATA
0017 GCAGCTTACCACGACCTTCTTCGCCTTAAAGCTCCTAGGCATTCACCATAGACTCTTATTACTTTGACCATATTTTTATCTTCCATCTCTATTTTGCCAATTTGTTTATG
0018 GCAGCTTACCACGACCTTCTTCGCCTTAAAGCTCCTAGGCATCCACCATAGACTCTTATTACTTTGACCATATTTTTATCTTCCATCTCTATTTTGCCAATTTGTTTATA
0019 GCAGCTTACCACGACCTTCTTCGCCTTAAAGCTCCTAGGCATCCACCATAGACTCTTATTACTTTGACCATATTTTTATCTTCCATCTCTATTTTGCCAATTTGTTTATA
0020 GCAGCTTACCACGACCTTCTTCGCCTTAAAGCTCCTAGGCATCCACCATAGACTCTTATTACTTTGACCATATTTTTATCTTCCATCTCTATTTTGCCAATTTGTTTATA
0021 GCAGCTTACCACGACCTTCTTCGCCTTAAAGCTCCTAGGCATCCACCATAGACTCTTATTACCTTGACCATATTTTTATCTTCCATCTCTATTTTGCCAATTTGTTTATA
0022 GCAGCTTACCACGACCTTCTTCGCCTTAAAGCTCCTAGGCATCCACCATAGACTCTTATTACTTTGACCATATTTTTATCTTCCATCTCTATTTTGCCAATTTGTTTATA
0023 GCAGCTTACCACGACCTTCTTCGCCTTAAAGCTCCTAGGCATTCACCATAGACTCTTATTACTTTGACCATATTTTTATCTTCCATCTCTATTTTGCCAATTTGTTCATA
0024 GCAGCTTACCACGACCTTCTTCACCTTAAAGCTCCTAGGCATTCACCATAGACTCTTATTACTTTGACCATATTTTTATCTTCCATCTCTATTTTGCCAATTTGTTCATA
0025 GCAGCTTACCACGACCTTCTTCGCCTTAAAGCTCCTAGGCATTCACCATAGACTCTTATTACTTTGACCATATTTTTATCTTCCATCTCTATTTTGCCAATTTGTTTATA
0026 GCAGCTTACCACGACCTTCTTCGCCTTAAAGCTCCTAGGCATTCACCATAGACTCTTATTACTTTGACCATATTTTTATCTTCCATCTCTATTTTGCCAATTTGTTTATA
0027 GCAGCTTACCACGACCTTCTTCGCCTTAAAGCTCCTAGGCATTCACCATAGACTCTTATTACTTTGACCATATTTTTATCTTCCATCTCTATTTTGCCAATTTGTTTATA
0029 GCAGCTTACCACGACCTTCTTCGCCTTAAAGCTCCTAGGCATTCACCATAGACTCTTATTACTTTGACCATATTTTTATCTTCCATCTCTACTTTGCCAATTTGTTTATG
0030 GCAGCTTACCACGACCTTCTTCGCCTTAAAGCTCCTAGGCATTCACCATAGACTCTTATTACTTTGACCATATTTTTATCTTCCATCTCTATTTTGCCAATTTATTTATA
0031 GCAGCTTACCACGACCTTCTTCGCCTTAAAGCTCCTAGGCATTCACCATAGACTCTTATTACTTTGACCATATTTTTATCTTCCATCTCTATTTTGCCAATTTGTTTATA
0032 GCAGCTTACCACGACCTTCTTCGCCTTAAAGCTCCTAGGCATTCACCATAGACTCTTATTACTTTGACCATATTTTTATCTTCCATCTCTATTTTGCCAATTTGTTTATG
```

```
          .250      .260      .270      .280      .290      .300      .310      .320      .330
PKo  CAACATAGAATAATATATATC-TTTGTTTAATCCATGTCAA--TATATATATTATTTTTTATA-TTATTTGAATGTTTTATTCAAATAATA
0001 CAACATAAAATAATATATATCTTTTGTTCAATCCATGTCAA--TATCTATTTTATTTTTTACA-TTATTTGAATAAAACATTCAAA-AACA
0002 CAACATAAAATAATATATATCTTTTGTTCAATCCATGTCAA--TATCTATTTTATTTTTTACA-TTATTTGAATAAAACATTCAAA-AACA
0003 CAACATAGAATAATATATATC-TTTGTTTAATCCATGTCAA--TATCTATTTTATTTTTTATATTTTTTTAAATCAAACATTCAAAAAA-A
0004 CAACATAGAATAATATATATC-TTTGTTTAATCCATGTCAA--TATATATATTATTTTTTATA-TTATTTGAATAAAACATTCAAATAATA
0005 CAACATAGAATAATATATATC-TTTGTTTAATCCATGTCAA--TATATATATTATTTTTTATA-TTATTTGAATAAAACATTCAAATAATA
0006 CAACATAGAATAATATATATC-TTTGTTTAATCCATGTCAA--TATATATATTATTTTTTATA-TTATTTGAATAAAACATTCAAATAATA
0007 CAACATAGAATAATATATATC-TTTGTTTAATCCATGTCAA--TATATATATTATTTTTTATA-TTATTTGAATAAAACATTCAAATAATA
0008 CAACATAAAATAATATATATC-TTTGTTTAATCCATGTCAA--TATATATATTATTTTTTATA-TTATTTGAATAAAACATTCAAATAATA
0009 CAACATAGAATAATATATATC-TTTGTTTAATCCATGTCAA--TATATATATTATTTTTTATA-TTATTTGAATAAAACATTCAAATAATA
0010 CAACATAGAATAATATATATC-TTTGTTTAATCCATGTCAA--TATATATTTTATTTTTTATA-TTATTTGAATAAAACATTCAAA-AACA
0011 CAACATAGAATAATATATATC-TTTGTTTAATCCATGTCAA--TATATATTTTATTTTTTATA-TTATTTGAATAAAACATTCAAA-AACA
0012 CAACATAGAATAATATATATC-TTTGTTTAATCCATGTCAA--TATATATTTTATTTTTTATA-TTATTTGAATAAAACATTCAAA-AACA
0013 CAACATAGAATAATATATATC-TTTGTTTAATCCATGTCAA--TATATATTTTATTTTTTATA-TTATTTGAATAAAACATTGAAA-AACA
0014 CAACATAGAATAATATATATC-TTTGTTTAATCCATGTCAA--TATATATTTTATTTTTTATA-TTATTCGAATAAAACATTCAAA-AACA
0015 CAACATAGAATAATATATATC-TTTGTTTAATCCATGTCAA--TATATATTTTATTTTTTATA-TTATTCGAATAAAACATTCAAA-AACA
0016 CAACATAGAATAATATATATC-TTTGTTTAATCCATGTCAATATATATATTTTATTTTTTATA-TTATTTGAATAAAACATTCAAATAATA
0017 CAACATAGAATAATATATATC-TTTGTTTAATCCATGTCAA--TATATATTTTATTTTTTATA-TTATTTGAATAAAACATTCAAAAAA-A
0018 CAACATAAAATAATATATATC-TTTGTTTAATCCATGTCAA--TATATATATTATTTTTTATA-TTATTTGAATGTTTTATTCAAATAATA
0019 CAACATAAAATAATATATATC-TTTGTTTAATCCATGTCAA--TATATATATTATTTTTTATA-TTATTTGAATGTTTTATTCAAATAATA
0020 CAACATAGAATAATATATATC-TTTGTTTAATCCATGTCAA--TATATATATTATTTTTTATA-TTATTTGAATGTTTTATTCAAATAATA
0021 CAACATAGAATAATATATATC-TTTGTTTAATCCATGTCAA--TATATATATTATTTTTTATA-TTATTTGAATGTTTTATTCAAATAATA
0022 CAACATAGAATAATATATATC-TTTGTTTAATCCATGTCAA--TATATATATTATTTTTTATA-TTATTTGAATGTTTTATTCAAATAATA
0023 CAACATAGAATAATATATATC-TTTGTTTAATCCATGTCAA--TATATATATTATTTTTTATA-TTATTTGAATGTTTTATTCAAATAATA
0024 CAACATAGAATAATATATATC-TTTGTTTAATCCATGTCAA--TATATATATTATTTTTTATA-TTATTTGAATGTTTTATTCAAATAATA
0025 CAACATAGAATAATATATATC-TTTGTTTAATCCATGTCAA--TATATATATTATTTTTTATA-TTATTTGAATGTTTTATTCAAATAATA
0026 CAACATAGAATAATATATATC-TTTGTTTAATCCATGTCAA--TATATATATTATTTTTTATA-TTATTTGAATGTTTTATTCAAATAATA
0027 CAACATAGAATAATATATATC-TTTGTTTAATCCATGTCAA--TATATATATTATTTTTTATA-TTATTTGAATGTTTTATTCAAATAATA
0029 CAACATAGAATAATATATATC-TTTGTTTAATCCATGTCAA--TATCTATTTTCTTTTTTATG-TTATTTGAATGGCTTATT---------
0030 CAACATAAAATAATATATATC-TTTGTTTAATGCATGTCAATATATATATTTTATTTTTTATG-TTATTTAAACAACACATTCAAA-AACA
0031 CAACATAAAATAATATATATC-TTTGTTTAATCCATGTCAA--TATATATTTCATTTTTTATG-TTATTTAAACAACACATTCAAA-AACA
0032 CAACATAAAGTAATATATATC-TTTGTTTAATACATGTCAA--TATATATTTCATTTTTTATG-TTATTTAAATAACACATTCAAA-AACA
```
