# Supplementary material for: Ability to cause erythema migrans differs between Borrelia burgdorferi sensu lato isolates
Source: Parasit Vectors. 2013 Jan 22;6:23. doi: 10.1186/1756-3305-6-23 (PMC3599126; doi:10.1186/1756-3305-6-23)
Supplement: Additional file 2: Figure S2 — Alignment of all identified OspC haplotypes with B. afzelii PKo (CP002934), REGION: 17065-17463. [file 1756-3305-6-23-S2.pdf]

Supplementary figure 2: Alignment of all identified OspC haplotypes with B. afzelii PKo (CP002934), REGION: 17065 - 17463

```
          .130      .140      .150      .160      .170      .180      .190      .200      .210      .220      .230
PKo  AATAAGCAAAAAAATTACAGATTCTAATGCATTTGTACTTGCTGTTAAAGAAGTTGAGACTTTGGTTTTATCTATAGATGAACTTGCTAAGAAAGCTATTGGTCAAAAAA
0001 AATAAGTAAAAAAATTACAGAATCTAACGCAGTTGTACTTGCTGTAAAAGAAGTTGAGACTTTGCTTGCATCTATAGACGAACTTGCT---AAAGCTATTGGACAAAAAA
0002 AATAAGTAAAAAAATTACAGATTCCAATACAGTTGTGTTGGCTGTAAAAGAAATTGAGACCTTGATTGCATCTATAGATGAACTTGCTAAGAAAGCTATTGGGCATTTGA
0003 AATAAGCAAAAAAATTACAGATTCTAATGCATTTGTACTTGCTGTTAAAGAAGTTGAGACTTTGGTTTCATCTATAGATGAACTTGCCAATAAAGCTATTGGTAAAAAAA
0004 AATAAGCAAAAAAATTACAGATTCTAATGCATTTGTACTGGCTGTTAAAGAAGTTGAGACTTTGCTTTCATCTATAGATGAACTTGCTAATAAAGCTATTGGTCAAAAAA
0005 AATAAGCAAAAAAATTACAGATTCTAATGCATTTGTACTGGCTGTTAAAGAAGTTGAGACTTTGGTTTCATCTATAGATGAACTTGCTACTAAAGCTATTGGTAAAAAAA
0006 AATAAGCAAAAAAATTACAGATTCTAATGCATTTGTACTGGCTGTTAAAGAAGTTGAGACTTTGGTTTCATCTATAGATGAACTTGCTACTAAAGCTATTGGTAAAAAAA
0007 AATAAGCAAAAAAATTACAGATTCTAATGCATTTGTACTGGCTGTGAAAGAAGTTGAGACTTTGCTTTCATCTATAGATGAACTTGCTACTAAAGCTATTGGTAAAAAAA
0008 AATAAGCAAAAAAATTACAGATTCTAATGCATTTGTACTTGCTGTTAAAGAAGTTGAGACTTTGGTTTCATCTATAGATGAACTTGCTGCTCAAGCTATTGGTAAAAAAA
0009 AATAAGCAAAAAAATTACAGATTCTAATGCATTTGTACTTGCTGTTAAAGAAGTTGAGACTTTGGTTTCATCTATAGATGAACTTGCTGCTCAAGCTATTGGTAAAAAAA
0010 AATAAGTAAAAAAATTACAGATTCTAATGCATTTGTACTGGCTGTGAAAGAAGTTGAAGCTTTGCTTTCATCTATAGATGAACTTGCTGCTCAAGCTATTGGTAAAAAAA
0011 AATAAGCAAAAAAATTACAGATTCTAATGCATTTGTACTGGCTGTTAAAGAAGTTGAGACTTTGGTTTCATCTATAAATGAACTTGCCACTCAAGCTATTGGTAAAAAAA
0012 AATAAGTAAAAAAATTACAGATTCTAATGCATTTGTACTTGCTGTTAAAGAAGTTGAGACTTTGGTTGCATCTATAGATGAACTTGCTACTAAAGCTATTGGTAAAAAAA
0013 AATAAGTAAAAAAATTACAAATTCTAATGCATTTGTACTTGCTGTTAAAGAAGTTGAGACTTTGGTTGCATCTATAGATGAACTTGCTACTAAAGCTATTGGTAAAAAAA
0014 AATAAGCAAAAAAATTACAGATTCTAATGCATTTGTACTTGCTGTTAAAGAAGTTGAGACTTTGGTTTCATCTATAGATGAACTTGCTACTAAAGCTATTGGTAAAAAAA
0015 AATAAGCAAAAAAATTACAGATTCCAATGCATTTGTACTAGCTGTTAAAGAAGTTGAGACTTTGGTTTCATCTATAGATGAACTTGCTGCTCAAGCTATTGGTAAAAAAA
0016 AATAAGCAAAAAAATTACAGATTCTAATGCATTTGTACTTGCTGTTAAAGAAGTTGAGACTTTGGTTTTATCTATAGATGAACTTGCTAAGAAAGCTATTGGTCAAAAAA
0017 AATAAGCAAAAAAATTACAGATTCTAATGCATTTGTACTGGCTGTGAAAGAAGTTGAGGCTTTGCTTTCATCTATAGATGAACTTGCT---AAATCTATTGGTAAAAAAA
0018 AATAAGCAAAAAAATTACAGATTCTAATGCAGTTGTACTGGTTGTGAAAGAAGTTGAGGCTTTGCTTTCATCTATAGATGAACTTTCT---AAAGCTATTGGTAAAAAAA
0019 AATAAGCAAAAAAATTACAGATTCTAATGCATTTGTACTGGCTGTGAAAGAAGTTGAGGCTTTGCTTTCATCTATAGATGAACTTGGCGCTAAAGCTATTGGTCAAAAAA
0020 AATAAGCAAAAAAATTACAGATTCCAATGCATTTGTACTAGCTGTTAAAGAAGTTGAGACTTTGGTTTCATCTATAGATGAACTTGCT---AGCGCTATTGGTCAAAAAA
0021 AATAAGCAAAAAAATTACAGATTCTAATTCATTTGTACTGGATGTTAAAGAAGTTGAGGCTTTGCTTTCATCTATAGATGAACTTGGT---AAAGCTATTGGTAAAAGAA
0022 AATAAGCAAAAAAATTACAGATTCTAATGCATTTGTACTGGCTGTGAAAGAAGTTGAGGCTTTGCTTTCATCTATAGATGAACTTGCT---AAAGCTATTGGTCAAAAAA
0023 AATAAGCAAAAAAATTACAGATTCTAATGCATTTGTACTGGCTGTGAAAGAAGTTGAGGCTTTGCTTTCATCTATAGATGAACTTGCT---AAAGGTATTGGTAAAAAAA
0024 AATAAGCAAAAAAATTACAGATTCTAATGCATTTGTACTGGCTGTTAAAGAAGTTGAGACTTTGGTTTCATCTATAGATGAACTTGCT---AAAGCTATTGGTAAAAAAA
0025 AATAAGCAAAAAAATTACAGATTCTAATGCATTTGTACTGGCTGTTAAAGAAGTTGAGACTTTGGTTTCATCTATAGATGAACTTGCT---AAAGCTATTGGTAAAAAAA
0026 AATAAGCAAAAAAATTACAGATTCTAATGCATTTGTACTGGCTGTTAAAGAAGTTGAGACTTTGGTTTCATCTATAGATGAACTTGCT---AAAGCTATTGGTAAAAAAA
0027 AATAAGCAAAAAAATTACAGATTCTAATGCATTTGTACTGGCTGTTAAAGAAGTTGAGACTTTGGTTTCATCTATAGATGAACTTGCT---AAAGCTATTGGTAAAAAAA
0028 AATAAGCAAAAAAATTACAGATTCTAATGCATTTGTACTCGCCGTTAAAGAAGTTGAGGCTTTGATTTCATCTGTAGATGAACTTGCT---AAGGCTATTGGTAAAAAAA
0029 AATAAGCAAAAAAATTACAGATTCTAATGCATTTGTACTGGCTGTGAAAGAAGTTGAGGCTTTGATCTCATCTATAGATGAACTTGCTAATAAAGCTATTGGTAAAAAAA
0030 AATAAGCAAAAAAATTACAGATTCTAATGCATTTGTACTAGCTGTGAAAGAAGTTGAGGCTTTGCTTTCATCTATAGATGAACTTGCT---AAAGGTATTGGTCAAAAAA
0031 AATAAGCAAAAAAATTACAGATTCTAATGCATTTGTACTAGCTGTGAAAGAAGTTGAGGCTTTGCTTTCATCTATAGATGAACTTGCT---AAAGGTATTGGTCAAAAAA
0032 AATAAGTAAAAAAATTACAGATTCAAATGCAATAGTACTAGCTGTTAAAGAAGTTGAGTCTCTACTTTTATCTATCGATGAGCTTGCT---AAAGGTATTGGTCAAAAAA
0033 AATAAGCAAAAAAATTACAGATTCTAATGCATTTGTACTAGCTGTGAAAGAAGTTGAGACTTTAATTTCATCTATAGATGAACTTGCTAATAAAGCTATTGGTCAAAAAA
0034 AATAAGCAAAAAAATTACAGATTCCAATGCAGTTGTACTAGCTGTGAAAGAAGTTGAGGCTTTGGTTTCATCTATAGATGAACTTGCT---AAAACTATTGGTAAAAAAA
0035 AATAAGCAAAAAAATTACAGATTCCAATGCAGTTGTACTAGCTGTGAAAGAAGTTGAGGCTTTGCTTTCATCTATAGATGAACTTGCT---AAAACTATTGGTAAAAAAA
0036 AATAAGCAAAAAAATTACAGATTCCAATGCAGTTGTACTAGCTGTGAAAGAAGTTGAGGCTTTGCTTTCATCTATAGATGAACTTGCT---AAAACTATTGGTAAAAAAA
0037 AATAAGCAAAAAAATTACAGATTCCAATGCAGTTGTACTAGCTGTGAAAGAAGTTGAGGCTTTGCTTTCATCTATAGATGAACTTGCT---AAAACTATTGGTAAAAAAA
0039 AATAAGCAAGAAAATTACAGATTCTAATGCAGTTGTACTGGCTGTGAAAGAAGTTGAAGCTTTGCTTTCATCTATAGATGAACTTGCCGCTCAAGCTATTGGTCAAAAAA
0040 AATAAGCAAAAAAATTACAGATTCTAATGAAGTTGTACTGGCTGTGAAAGAAGTTGAGGCTTTGCTTTCATCTATAGATGAAATTGCCACTCAAGCTATTGGTAAAAAAA
0041 AATAAGCAAAAAAATTACAGATTCTAATGCATTTGTACTGGCTGTGAAAGAAGTTGAAGCTTTGCTTTCATCTGTAGATGAACTTGCC---AAAGCTATTGGTAAAAAGA
0042 AATAAGCAAAAAAATTACAGATTCTAATGCAGTTGTACTGGCTGTGAAAGAAGTTGAAGCTTTGCTTTCATCTATAGATGAACTTGCT---AAAGCTATTGGTCAAAAAA
0043 AATAAGTAAAAAAATTACAGATTCTAATGCATTAGTACTAGCTGTGAAAGAAGTTGAGACTTTGGTTTCATCTATAGATGAAATTGGTACTAAAGCTATTGGTCAAAAAA
0044 AATAAGTAAAAAAATTACAGATTCTAATGCATTAGTACTAGCTGTGAAAGAAGTTGAGACTTTGATTTCATCTATAGATGAACTTGCT---AAAGCTATTGGTAAAAAAA
0045 AATAAGTAAAAAAATTACAGATTCTAATGCATTAGTACTGGCTGTGAAAGAAGTTGAGACTTTGGTTTCATCTATAGATGAACTTGCT---AAAGCTATTGGTCAAAAAA
0046 AATAAGTAAAAAAATTATAGATTCTAATGCATTAGTACTGGCTGTAAAAGAAGTTGAGACTTTGGTTTCATCTATAGATGAAATTGGTGCTAAAGCTGTTGGTCAAAAAA
```

```
           .240      .250      .260      .270      .280      .290      .300      .310      .320      .330      .340
PKo  TAGACAATAATAATGGTTTAGCTGCTTT---AAATAATCAGAATGGATCGTTGTTAGCAGGAGCCTATGCAATATCAACCCTAATAACAGAAAAATTGAGTAAATTGA--
0001 TAGAATCAAATGGCGGTTTAAATGCTGA---TGGAAATCAAAACGGATCATTAATATCAGGAGCATATTCAATTTCAAAATTAATAAAACAAAAATTGAGTATATTAA--
0002 TAGTCCAAAAT---GGTTTAAATGCCAG---TCCGGCTCAAAACGGATCATTGTTGGCGGGAGCCTACGTAATATCAACCCTAATAACACAAAAATTAAGTGGATTAA--
0003 TACAACAAAAT---GGTTTAGGCGCCGA---AGCGAATCGCAACGAATCATTGTTAGCAGGAGTTCATGAAATATCAACACTAATAACAGAAAAATTAAGTAAATTGA--
0004 TAGACCAAAATAATGGTTTAGGCGCCAA---TGCGGATAAAAACGGATCATTGTTAGCAGGAGCTTATGCAATATCAACTCTAATAACAGAAAAATTGAGTAAATTGA--
0005 TACAACAAAATAATGGTTTAGGCGCCAA---TGCGGATAAAAACGGATCATTGTTAGCAGGAGCTTATGCAATATCAACCCTAATAACAGAAAAATTAAAGGCATTGA--
0006 TACAACAAAATAATGGTTTAGGTGCCAA---TGCGGATAAAAACGGATCATTGTTAGCAGGAGCTTATGCAATATCAACCCTAATAACAGAAAAATTAAAGGCATTGA--
0007 TACATCAAAATAATGGGCTAGATACTGA---AGCGAATCGCAACGAATCATTGTTAGCAGGAGCTTATGCGATATCAAACCTAATAACAGAAAAATTGAGTAAATTGA--
0008 TACAAAACAAT---GGTTTGACTGCCGA---ACAGAATCAAAACGGATCATTGTTGGCCGGAGCCTATGCAATATCAGCCCTAATAACAAAAAAATTAGATGAATTGACC
0009 TACAAAACAAT---GGTTTGACTGCCGA---ACAGAATCAAAACGGATCATTGTTGACCGGAGCCTATGCAATATCAGCCCTAATAACAAAAAAATTAGATGAATTGACC
0010 TACAAAACAAT---GGTTTGACTGCCGA---ACAAAATCAAAACGGATCATTGTTAGCCGGAGCCTATGCAATATCAGCCCTAATAACAAAAAAATTAGATGAATTGACC
0011 TAGACCAAAAT---GGTTTGGGTGCTTT---ACAGAATCAAAACGGATCATTGTTAGCAGGAGCCTATGCAATATCAGTCCTAATAACAAAAAAATTAGATGAATTGATC
0012 TAAAAAATGATGGCACTTTAGATAACGA---AGCAAATCACAACGGATCATTGTTAGCAGGAGCCTATGCAATATCAACTCTAATAACACAAAAATTAAGTGTATTGA--
0013 TAAAAAATGATGGCACTTTAGAGAACGA---AGCAAATCACAACGGATCATTGTTAGCGGGAGCTTATGCAATATCAAATCTAATAAAACAAAAATTAGATGGATTGA--
0014 TACAACAAAACAATGGTTTAGCTGCTGA---AGCAGATAAAAACGGATCATTGTTAGCGGGAGCTTATGCAATATCAAATTTAATAAAACAAAAATTAGATGGATTGA--
0015 TACAAAACAAT---GGTTTAGCTGCTGA---AGCAAATAAAAACGGATCATTGTTAGCGGGAGCTTATGCAATATCAAATCTAATAAAACAAAAATTAGATGGATTGA--
0016 TAGACAATAATAATGGTTTAGCTGCTTT---AAATAATCAGAATGGATCGTTGTTAGCAGGAGCCTATGCAATATCAACCCTAATAACAGAAAAATTGAGTAAATTGA--
0017 TAGATGCAAAC---GGTTTGGGTGCTGA---TGCAAATCAGAACGGACCATTATTAGCAGGAGCCTATGCAATATCAAACTTAATATCAGAAAAATTAAGTAAATTGA--
0018 TAAGAAATGATGGTACTTTAGATAACGA---AGCAAATCGAAACGAATCATTGATAGCAGGAGCTTATGAAATATCAAAACTAATAACACAAAAATTAAGTGTATTGA--
0019 TAGGTCAAAAT---GGTTTAGAAGTTGATGGGGGGGGTCACAACACCGCATTGTTAGCAGGAGCTTATGCAATATCAGCCCTAATAAAACAAAAATTAGATGTATTAC--
0020 TAGACCAAAAT---GGTTTAGCTGCTGATGCGGCTGATCACAACACCTCATTGTTAGCAGGAGCCTATGCAATATCAGCCCTAATAAAACAAAAATTAGATGGATTGA--
0021 TACAACAAAAT---GGTTTAGCTGCTGATGCGCCTAATCAAAACGGATCATTGTTAGCAGGAGCCCATGAAATATCAATTCTAATAAAACAAAAATTAGATGGATTGA--
0022 TAGATCAAAATAATGGTTTAGCTGCTGCTACTCAGGATAAAAACACCTCATTGTTAGCAGGAGCCTATGCAATATCAGCCCTAATAAAACAAAAATTAGATGGATTGC--
0023 TAGATCAAAATAGTGGTTTAGCTGCTGCTACTCAGAATAAAAACACCTCGTTGTTAGCAGGAGCCTATGCAGTATCAGCTCTAATAAAACAAAAATTAGATGGATTGC--
0024 TAGATAACAATAATGGTTTAAGTGCTAA---TGCGAATTTAAACACCTCGTTGTTAGCAGGAGCCTATGCAATATCAACCCTAATAAAACAAAAATTAGATGGATTGA--
0025 TAGATAACAATAATGGTTTAAGTGCTAA---TGCGAATTTAAACACCTCGTTGTTAGCAGGAGCCTATGCAATATCAACCCTAATAAAACAAAAATTAGATGGATTGA--
0026 TAGATAACAATAATGGTTTAAGTGCTAA---TGCGAATTTAAACACCTCATTGTTAGCAGGAGCCTATGCAATATCAACTCTAATAACACAAAAATTAAGTGTATTGA--
0027 TAGATAACAATAATGGTTTAAGTGCTAA---TGCGAATTTAAACACCTCGTTGTTAGCAGGAGCCTATGCAATATCAACCCTAATAAAACAAAAATTAGAGGGATTGA--
0028 TAGATAACAATACTGGTTTAAGTGCTAA---TCAGAATCATAACACTTCATTGTTAGCAGGAGCCTATTCAATATCAACCCTAATAACAGAAAAATTAAGTAAATTAA--
0029 TAAATCAAAAT---GGTTTAGATGCTGA---TGCTAATCACAACGGATCATTGTTAGCAGGAGCCCATGCAATATCAACTCTAATAAAACAAAAACAGATGGATTGA--
0030 TAGATGCAAAT---GGTTTAAATGTTGCTGAAGCGGATAAAAACACCTCATTGTTAGCAGGAGCCTATTCAATATCAACCCTAATAACAAAAAAGTTAGATGAATTGATC
0031 TAGATGCAAAT---GGTTTAAATGTTGCTGAAGCGGATAAAAACACCTCATTGTTAGCAGGAGCCTATTCAATATCAACCCTAATAACAAAAAAGTTAGATGAATTGATC
0032 TAGATGCAAAT---GGTTTAAGTGCTGCTGATGCGAATAAAAACACCTCATTGTTAGCAGGAGCCTATTCAGTATCAAATCTAATAACACAAAAATTAAGTGCACTAA--
0033 TAGGTCAAAAT---GGTTTGGCTGTCGA---AGCTGATAAAAACAACTCATTGTTAGCAGGAGCCTATGCAGTATCAAGTCTAATAACACAAAAATTAAGTGCATTAA--
0034 TAGAGGCAAAT---GGTTTGGGTAACGA---AGCGGATAAAAACACCTCATTGTTGGCGGGAGCCTATTCAATATCAAGCCTAATAACAAAAAAATTAGAGGGATTGATC
0035 TAGAGGCAAAT---GGTTTGGGTAACGA---AGCGGATAAAAACACCTCATTGTTGGCGGGAGCCTATTCAATATCAAGCCTAATAACAAAAAAATTAGAGGGATTGATC
0036 TAGAGGCAAAT---GGTTTGGGTAACGA---AGCGGATAAAAACACCTCATTGTTGGCGGGAGCCTATTCAATATCAAGCCTAATAACAAAAAAATTAGAGGGATTGATC
0037 TAGAGGCAAAT---GGTTTGGGTAACGA---AGCGGATAAAAACACCTCATTATTGGCGGGAGCCTATTCAATATCAAGCCTAATAACAAAAAAATTAGAGGGATTGATC
0039 TAGGTCAAAAT---GGTTTAACTGCCGA---AGCGAATTACAACAACTCATTGTTAGCAGGAGCCTATGCAATATCAAGTCTAATAACAAAAAAATTAGAGGAATTGATC
0040 TAGATGGAAAT---GGTTTAACTGCTGA---TGGGGATCACAACAATTCATTGTTAGCAGGAGCCTATGCAATATCAACCCTAATAACAAAAAAATTAGATGGATTGA--
0041 TACATCAAAATAATGGTTTAGATACTCT---GTCAAATCAAAACGGATCATTGTTAGCAGGAGCCTATGCAATATCAACCCTAATAACAAAAAAATTAGATGGATTGA--
0042 TAGATCGAAATAATGGTTTAGCTGTCGA---AGCGAATTTTAACACCTCATTGTTAGCAGGAGCCTATACAATATCAACCCTAATAACAAAAAAATTAGATGAATTGATC
0043 TAGGTCAGAAT---GGTTTAGAAGCTGA---TGCAAATAAAAACACATTATTGTTAGCAGGAGCTTATGCAATAGCAGCCCTAATAACAGAAAAATTAACAGTATTAA--
0044 TACAGGCAGCT---GGTTTAGAAGCTGA---AGCGAATAAAAACACATCATTGTTAGCAGGAGCTTATGCAATATCAACCCTAATAACAGAAAAATTAGGTAAATTGA--
0045 TAGGTCAGAAT---GGTTTAGATGTTAA---CGCGGATAGAAACACATCATTGTTAGCAGGAGCTTATGCAATATCAACCCTAATAACAGAAAAATTAAATGGATTGA--
0046 TAGATCAAAATGCTGGTTTATCCGTCCA---AGCAAATCAGAACGGATCATTGTTAGCAGGAGCTTATGCAATAGCAACCCTAATAACAGACAAATTAACTGGATTGA--
```

```
           .350      .360      .370      .380      .390      .400      .410      .420      .430      .440      .450
PKo  -AAAATTTAGAAGAATTAAAGACAGAAATTGCAAAGGCTAAGAAATGTTCCGAAGAATTTACTAATAAACTAAAAAGTGGTCATGCAGATCTTGGC---------AAACA
0001 ----ATTCAGAAGCATTAAAGGAAAAAATTGATGCGGCTAAGAAATGTTCTGAAGCATTTACTGACAAATTAAAAAGTGAGCACGCAAGTCTTGGT---------AAAAA
0002 -AAGCTTCAGAAGACTTAAAAGAAAAAATTACAAAGGCTAAGGAATGTTCTGAGAAATTTACTGATAAACTAAAAAGTGAAAATGTAGCGCTTGGCAAA------CAGGA
0003 -AAAATTCAGGAGAATTAAAGGCAAAAATTGAAGATGCTAAGAAATGTTCTGAAGAATTTACTAATAAACTAAGAGTTAGTCATGCAGATCTTGGT---------AAACA
0004 -AAAATTCAGAAGAATTAAAGGCAGAAATTGAGGAGGCTAAGAAATGTTCCGAAGACTTTACTAAAAAACTAAAAGATAATCACACAGATCTTGGT---------ATAGA
0005 -AAAATTCAGGAGAATTAAAGGCAAAAATTGAAGATGCTAAGAAATGTTCTGAAGATTTTACTAAAAAACTAGCTGCTGGGCATGCACAGCTTGGT---------ATAGA
0006 -AAAATTCAGGAGAATTAAAGGCAAAAATTGAAGATGCTAAGAAATGTTCTGAAGATTTTACTAAAAAACTAGCTGCTGGGCATGCACAGCTTGGT---------ATAGA
0007 -AAAATTCAGGAGAATTAAATAAAGAAATTGCAGATGCTAAGAAATGTTCTGAAGAATTTACTAAAAAACTAAAAGATAGTCATGGCAGCTTGGT---------ATACA
0008 AAAAATTCAGGAGAATTAAAAGGAGAAGTTGAAAAAGCTAAGAAATGTTCCGAAGAATTTACTAATAAACTAAAAGGTGGTCATGCAGAGCTTGGA---------CTTGC
0009 AAAAATTCAGGAGAATTAAAAGGAGAAGTTGAAAAAGCTAAGAAATGTTCCGAAGAATTTACTAATAAACTAAAAGGTGGTCATGCAGAGCTTGGA---------CTTGC
0010 AAAAATTCAGGAGAATTAAAAGGAGAAGTTGAAAAAGCTAAGAAATGTTCCGAAGAATTTACTAATAAACTAAAAGGTGGTCATGCAGAGCTTGGA---------CTTGC
0011 AAAAATTCAGGAGAATTAAAAGGAGAAGTTGAAAAGACCAAAAACTGTTCTGTAGCATTTACTAATAAATTAAAAGAGAAGCACCAAGAACTTGGAATTGCTGC---TGG
0012 ----ATTCAGAAGAATTAAAGGCAGAAATTGTAAAGGCTAAGAAATGTTCCGAAGACTTTACTAAAAAACTAAAAGATAAGCACACAGAACTTGGT---------AAACA
0013 -AAGGTTTAGAAGGATTAAATAAGGAAATTGCGGAGGCCAAGAACTGTTCTGAAGCATTTACTAAAAAACTAAAAGAGAAGCACACAGATCTTGGG---------AAAGA
0014 -AAGGTTTAGAAGGATTAAATAAGGAAATTGCGGAGGCCAAGAAATGTTCCGAAGCATTTACTAAAAAGCTACAAGATAGTAACGCAGATCTTGGG---------AAACA
0015 -AAGGTTTAGAAGGATTAAATAAGGAAATTGCGGAGGCCAAGAAATGTTCCGAAGCATTTACTAAAAAGCTACAAGATAGTAACGCAGATCTTGGG---------AAACA
0016 -AAAATTTAGAAGAATTAAAGACAGAAATTGCAAAGGCTAAGAAATGTTCCGAAGAATTTACTAATAAACTAAAAAGTGGTCATGCAGATCTTGGC---------AAACA
0017 -AAAATTCAGAAGCATTGAAAGAAAAAATTGAAAAGACTAAAGAATGTTCCAAAAAATTTACTGACAAACTAAAAAGTGGTCATGCGGACCTTGGA-----------CA
0018 ----ATTCAGAAGAATTAAAGGAAAAAATTAAAGAGGCTAAGGATTGTTCCGAAAAATTTACTACTAGGCTGAGAGATAGTCATGCAGAGCTTGGT---------ATACA
0019 -AAGGTCCAGAAGGGTTAAATAAAGAAATTGATGAGGCTAAGAAATGTTCTGAAGCATTTACCAATAAATTAAAAAGTAGTCATGTAGATCTTGGAGTGGCGGC---TGG
0020 -AAGGTCCAGAAGGGTTAAATAAAGAAATTGATGAGGCTAAGAAATGTTCTGATGCATTTACTAATAAATTAAAAAATAGTCATGCAGAGCTTGG---GGCGGCGAATGG
0021 -AAGGTCTAGAAGGATTAAATAAAGAAATTGAAGCAGCTAAGAAATGTTCTGCAGCATTTACCAATAAATTAAAAAGTAGTCATGCAGATCTTGGAGCGGCGGC---TGG
0022 -AAGGTCCAGAAGGGTTAAATAAAGAAATTGAAGCGGCTAAGAAATGTTCTGAAGCATTTACTAATAAATTAAAAGAGAAGCACCAAGACCTTGGAGTGGCG---AATGG
0023 -AAGGTCCAGAAGGGTTAAATAAAGAAATTGAAGCGGCTAAGAAATGTTCTGAAGCATTTACTAATAAATTAAAAGAGAAGCACCCAGAACTTGGAGTGGCGGG---TGG
0024 -AAGGTCTAGAAGGATTAAAGGAAAAAATTGAAAAGGCTAAGAACGCTTCTGCAGCATTTACTAATAAATTAAAAAATAGTCATGCAGAGCTTGGAGTGGCGGGGAATGG
0025 -AAGGTCTAGAAGGATTAAAGGAAAAAATTGAAAAGGCTAAGAACGCTTCTGCAGCATTTACTAATAAATTAAAAAATAGTCATGCAGAGCTTGGAGTGGCGGGGAATGG
0026 ----ATTCAGAAGGATTAAAGGAAAAAATTGAAAAGGCTAAGAACGCTTCTGCAGCATTTACTAATAAATTAAAAAATAGTCATGCAGAGCTTGGAGTGGCGGGGAATGG
0027 -AAGGTCTAGAAGGATTAAAGGAAAAAATTGAAAAGGCTAAGAACGCTTCTGCAGCATTTACTAATAAATTAAAAAATAGTCATGCAGAGCTTGGAGTGGCGGCGAATGG
0028 -AAAATTTAGAAGGGTTAAAAGCAGAGATTGCAGAAGCTAAGAAATGTTCTGAAGACTTTACTAAAAAACTAAAAGATAATCATGCAGATCTTGGAGTGGCGGGGAATGG
0029 -AAGATCTAGAAGGGTTAAGTAAAGAAATTGCAAAGGTGAAGGAATGTTCCGATAAATTTACTAAAAAGCTAACAGATAGTCATGCACAGCTTGGAGCAGT---TGGTGG
0030 AAAAATTCAGGAGAGTTAAAAGGAGAAGTTGAAAATGCTAAAAACTGTTCTGATGCATTTACTAATAAATTAAAAGATGTTCGTGCACAACTTGGTGTGCAG---AATGG
0031 AAAAATTCAGGAGAGTTAAAAGGAGAAGTTGAAAATGCTAAAAACTGTTCTGATGCATTTACTAATAAATTAAAAGATGTTCGTGCACAACTTGGTGTGCAG---AATGG
0032 -AAGATTCAGGAGAATTAAAGGCAGAAATTGAAGAGGCTAAGAAATGTTCTGCAGCATTTACTAATAAATTAAAAAGTAGTAACGCAGATCTTGGTGTGCAG---AATGG
0033 -AAGATTCAGGAGAATTAAAGGCAGAGATTGCAGAAGCTAAGAAATGGTCTGCAGAATTTACTAATAAATTAAAAAGTAGTAACGCAGAGCTTGGAGCGGCG---AATGG
0034 AAAAATTCAGGAGAATTAAAGACAGAAGTTGAAAAGGCTAAGAACGCTTCTGCAGCATTTACTAATAAATTAAAAACTAGTCATGCACAACTTG---GGGCGGCGAATGG
0035 AAAAATTCAGGAGAATTAAAGACAGAAGTTGAAAAGGCTAAGAACGCTTCTGCAGCATTTACTAATAAATTAAAAACTAGTCATGCACAACTTG---GGGCGGCGAATGG
0036 AAAAATTCAGGAGAATTAAAGACAGAAGTTGAAAAGGCTAAGAACGCTTCTGCAGCATTTACTAATAAATTAAAAAATAGTCATGCACAACTTG---GGGCGGCGAATGG
0037 AAAAATTCAGGAGAATTAAAGACAGAAGTTGAAAAGGCTAAGAACGCTTCTGCAGCATTTACTAATAAATTAAAAACTAGTCATGCACAACTTG---GGGCGGCGAATGG
0039 AAAAATCCAGGAGAATTAAAGCAGAGATTTCAGAAGCTAAGAAATGTTCTGAAGCATTTACTAAAAAACTAAAAGATAGTAATGCACAGCTTGGTATGCAG---AATGG
0040 -AAGGTTCAGAAGGATTAAAAGCAGAAATTGCAAAGGCTAAGAAATGTTCCGAAGCATTTACTACTCAATTAAAAGCTAGTCATGCACAACTTGGAGCAGCGGC---TGG
0041 -AAGGTTCAGAAGGATTAAAAGCAGAAATTGCAGAAGCTAAGAAATGTTCTGAAGACTTTACTAAAAAACTAAAAGAGAAGCATACAGAACTTGGA---------GTTGC
0042 AAAAATTCAGGAGAATTAAAAGGAGAAGTTGAAAAGGCTAAAAACTGTTCTGAAGCATTTACTAATAAATTAAAAGAGAAGACCCAAGAACTTGCAGTGGCGGC---TGG
0043 ----ATTCAGAAGAATTAAAGGACAAAATTGATGAAGCTAAGAAATGTTCTGAAGCATTTACTACAAAACTAAAACAAAGTCATGCAGAGCTTGGTGTAGCTGCTGGTGC
0044 ----ATTCAGAAGAATTAAAAGACAAAATTGATGCGGCTAAGAAATGTTCTGAAGATTTTAGTGCAAAACTAAAACAAAGTCATGCAGAGCTTGGTGTAGCTGC---TGG
0045 ----ATTCAGAAGAATTAAAGGGAAAGCTTGATGAAGCTAAGAGATTTTCTGAAGCATTTACTACAAAACTAAAAACTGAGAATGTAGTGCTTGGTGTGGCTGCTAATGG
0046 ----AGTCAGAAGAATTAAAGGACAAAATTGATGAAGCTAAGAAATGTTCTGAAGATTTTACTACAAAACTAAAAAGTGAGCATGCAGCGCTTGGTATAGC---------
```

```
          .460      .470      .480      .490      .500      .510      .520      .530
PKo  GGATGCTACCGATGATCATGCAAAAGCAGCTATTTTAAAAACACATGCAACTACCGATAAAGGTGCTAAAGAATTTAAAGATTT
0001 AGATGCTACTGATGATGATGCAAAAAAGGCTATTTTAAAAACAAACGTAGATAAAACTAAGGGTGCTGATGAGCTTATAAAATT
0002 TGCTAGTGATGATGA---TGCAAAAAAAGCTATTTTAAAAACACATAATGATATAACTAAGGGTGCTAAAGAACTTAAAGAGTT
0003 AGGTGTTAATGACGATGATGCAAAAAAAGCTATTTTAAAAACAAATGCAGATAAAACTAAAGGTGCTGAAGAACTTGGAAAGTT
0004 TAATGCTACCGATGATAATGCAAAAAAAGCTATTTTAAAAACAAATGCAGATAAAACCAAAGGTGCTGAAGAACTTGAAAAGTT
0005 CGGAGCTACTGATAATGATTCAAAAGAAGCAATTTTGAAAACAAATGGGACTAAAACTAAGGGTGCTGAAGAACTTGTAAAGTT
0006 CGGAGCTACTGATAATGATTCAAAAGAAGCAATTTTGAAAACAAATGGGACTAAAACTAAGGGTGCTGAAGAACTTGTAAAGTT
0007 AAATGTTACTGATGATAATGCAAAAAAAGCTATTTTAAAAACAAATGCGGACAAGGCTCTGGGTGCCGGTGAACTTGAAAAGCT
0008 TGCTGCTACTGATGAAAATGCAAAAAAAGCCATTTTAAAAACAAATGGAACTAAAGATAAGGGGGCTGAAGAACTTGAAAAGTT
0009 TGCTGCTACTGATGAAAATGCAAAAAAAGCCATTTTAAAAACAAATGGAACTAAAGATAAGGGGGCTGAAGAACTTGAAAAGTT
0010 TGCTGCTACTGATGAAAATGCAAAAAAAGCCATTTTAAAAACAAATGGAACTAAAGATAAGGGGGCTGAAGAGCTTGAAAAGTT
0011 TGCTGCTACTGATGTTAATGCAAAAGCAGCTATTTTAAAAACAGATACTACTAAAGATAAGGGTGCTACTGAGCTTGGAGAGTT
0012 GGATGCTAATGATGATGATGCAAAAAAAGCTATTTTAAAAACAAATGGCGATAAAACTTTGGGTGCTGCTGAACTTGAAAAGCT
0013 GAATGCTACCGATGAAGATGCAAAAAAAGCTATTTTAAAAACAGATGCTACTAAAGATAAGGGTGCTGCTGAACTTGAAAAGCT
0014 TGATGCTACTGATGCTGATGCAAAAAAAGCTATTTTAAAAACAGATGCTACTAAAGATAAGGGTGCTAAAGAACTTGAAGAGTT
0015 TGATGCTACTGATGCTGATGCAAAAAAAGCTATTTTAAAAACAAATGGAACTAAAGATAAGGGGGCTGAAGAACTTGAAAAGTT
0016 GGATGCTACCGATGATCATGCAAAAGCAGCTATTTTAAAAACACATGCAACTACCGATAAAGGTGCTAAAGAATTTAAAGATTT
0017 AGGTGCTACTGATGTAAATGCAAAAAAAGCTATTTTGAAAACAGATGCAACTAAAGATAAGGGTGCTAAAGAGCTTGAAGATTT
0018 AAACGTTCAGGATGATAATGCAAAAAGAGCTATTTTAAAAACACATGGGAATAAAGACAAGGGTGCTAAAGAACTTAAAGAGTT
0019 TGCTACTACTGATGATAATGCAAAAAGAGCTATTTTAAAAACACATGGGAATGACAATAAGGGTGGTAAAGAACTTAAAGACTT
0020 TGCTACTACTGCTGATAATGCAAAAAGAGCTATTTTAAAAACACATGCGAATGAGGACAAGGGTGCTAAAGAACTTAAAGCCTT
0021 TGCTACTACTGATGATAATGCAAAAAGAGCTATTTTAAAAACACATGGGAATGATGAAAGGGGTCTTAAAGAACTTAAAGAGTT
0022 TGAGACTACTGATAATAATGCAAAAGCAGCTATTTTAAAAACACATGGGACTGAGGACAAGGGTGTTAAAGAACTTAAAGATTT
0023 TGATACTACTGATGATAATGCAAAAGCAGCTATTTTAAAAACACATGCTACTGAGGATAAGGGTGTTGAAGATCTTGAAAAGTT
0024 TGCTACTACTGATGAAAATGCACAAAAAGCTATTTTAAAAACAAATGCGAATAACGATAAGGGTGCTAAAGAACTTAAAGAGTT
0025 TGCTACTACTGATGAAAATGCACAAAAAGCTATTTTAAAAACAAATGGAAATAACGATAAGGGTGCTAAAGAACTTGAAGAGTT
0026 TGCTACTACTGATGAAAATGCACAAAAAGCTATTTTAAAAACAAATGCGAATAACGATAAGGGTGCTAAAGAACTTAAAGAGTT
0027 TGGTGCTACTGATGCTCATGCAAAAGAAGCTATTTTGAAGTCAAATCCTACTAAAGATAAGGGTGTTACAGAGCTTGAAGAGTT
0028 AGCTTCTACTGATGAAAATGCACAGAAAGCTATTTTAAAAACAAATGCGATTGTCGATAAGGGTGCTAAAGAACTTAAAGAGTT
0029 TGCTATTAATGATGATCGTGCAAAAGAAGCTATTTTAAAAACACATGGGACTAACGATAAGGGTGCTAAAGAACTTAAAGAGTT
0030 TGGTGCTACTGATGATCATGCAAAAGCAGCTATTTTAAAGTCAAATCCTACTAAAGATAAGGGTGTTGATGAGCTTGAAAAGTT
0031 TGGTGCTACTAATGATCATGCAAAAGCAGCTATTTTAAAGTCAAATCCTACTAAAGATAAGGGTGTTGATGAGCTTGAAAAGTT
0032 TGGTGCTACTAATGATCATGCAAAAGCAGCTATTTTAAAGTCAAATAATACTAAAGATAAGGGTGTTGATGAACTTGAAAAGTT
0033 TGGTGCTACTGATGCTCATGCAAAAGCAGCTATTTTAAAGTCAAATGTTGCTAAAGATAAGGGTGTTGATGAGCTTGACAAGCT
0034 TGGTGCTACTGATGCTCATGCAAAAGAAGCTATTTTGAAGTCAAATCCTACTAAAGATAAGGGTGTTACAGAGCTTGAAGAGTT
0035 TGGTGCTACTGATGCTCATGCAAAAGAAGCTATTTTGAAGTCAAATCCTACTAAAGATAAGGGTGTTACAGAGCTTGAAGAGTT
0036 TGGTGCTACTGATGCTCATGCAAAAGAAGCTATTTTGAAGTCAAATCCTACTAAAGATAAGGGTGTTACAGAGCTTGAAGAGTT
0037 TGGTGCTACTGATGCTCATGCAAAAGAAGCTATTTTGAAGTCAAATCCTACTAAAGATAAGGGTGTTACAGAGCTTGAAGAGTT
0039 TGCTGCTACTGATGCTCATGCAAAAGCAGCTATTTTAAAAACAGATGCTACTAAGGATAAGGGTGCTACTGAGCTTGGAGAGTT
0040 TGCTGCTACTAGTGAAAATGCAAAAAAAGCTATTTTAAAAACAAATGGTACTAAGGATAAGGGTGCTGCTGAGCTTGAAAAGTT
0041 TGCTGCTACTGATGATAATGCAAAAAAAGCTATTTTAAAAGCAAATGGGGATAAGACTTTAGGTGTTGAAGAGCTTGAAAAGTT
0042 TGCTGCTACTGATATTGATGCAAAAAAAGCTATTTTAAAAACAAATAGGGACAAGGACCTAGGTGCTGATGAACTTGGCAAGTT
0043 TGCTACTACTGATGTGAATGCAAAAACCGCTATTTTAAAAACAGATGCGGGTG---ATAGGGGTGTTAAAGAGTTTAACGCGCT
0044 TACTGCTAGTGATGAGAATGCAAAAAAAGCTATTTTAAAAACAAATAATCCGGGTGATAAGGGTGTTCAAGAGCTTAAAAAGCT
0045 TGCTTCTACTGATGCTGATGCAAAAAAAGCTATTTTAAAAACAGATGCTGTGGGTGATAAGGGCGTTCAAGAGCTTAAAAGTCT
0046 TGCTGCTACTGATGCGCATGCAAAAAACGCTATTTTAAAAACAGATAATGGCG---ATAAGGGTGTTAAAGAGTTTAACAAGCT
```
